# Long Non-Coding RNAs as Mediators of Tumor Microenvironment and Liver Cancer Cell Communication

**DOI:** 10.3390/ijms19123742

**Published:** 2018-11-24

**Authors:** Yang-Hsiang Lin, Meng-Han Wu, Chau-Ting Yeh, Kwang-Huei Lin

**Affiliations:** 1 Department of Biochemistry, College of Medicine, Chang Gung University, Taoyuan 333, Taiwan; yhlin0621@cgmh.org.tw (Y.-H.L.); snoopy740621@yahoo.com.tw (M.-H.W.); 2 Liver Research Center, Chang Gung Memorial Hospital, Linkou, Taoyuan 333, Taiwan; chauting@cgmh.org.tw; 3 Research Center for Chinese Herbal Medicine, College of Human Ecology, Chang Gung University of Science and Technology, Taoyuan 333, Taiwan

**Keywords:** tumor microenvironment, hepatocellular carcinoma, long non-coding RNA, therapeutic target

## Abstract

The tumor microenvironment is an important concept that defines cancer development not only through tumor cells themselves but also the surrounding cellular and non-cellular components, including stromal cells, blood vessels, infiltrating inflammatory cells, cancer stem cells (CSC), cytokines, and growth factors, which act in concert to promote tumor cell survival and metastasis. Hepatocellular carcinoma (HCC) is one of the most common and aggressive human malignancies worldwide. Poor prognosis is largely attributable to the high rate of tumor metastasis, highlighting the importance of identifying patients at risk in advance and developing novel therapeutic targets to facilitate effective intervention. Long non-coding RNAs (lncRNA) are a class of non-protein coding transcripts longer than 200 nucleotides frequently dysregulated in various cancer types, which have multiple functions in widespread biological processes, including proliferation, apoptosis, metastasis, and metabolism. lncRNAs are involved in regulation of the tumor microenvironment and reciprocal signaling between cancer cells. Targeting of components of the tumor microenvironment or cancer cells has become a considerable focus of therapeutic research and establishing the effects of different lncRNAs on this network should aid in the development of effective treatment strategies. The current review provides a summary of the essential properties and functional roles of known lncRNAs associated with the tumor microenvironment in HCC.

## 1. Introduction

The tumor microenvironment is an important concept that defines cancer development not by tumor cells alone but also the surrounding cellular and non-cellular components, including stromal cells, blood vessels, infiltrating inflammatory cells, cancer stem cells (CSC), cytokines, and growth factors, which act together to promote tumor cell survival and metastasis [1]. Stromal cells are recruited and activated in tumor progression, in turn, triggering downstream signals that promote invasion to distant organs. The surrounding environment appears to be a crucial partner for tumor cells and provides several of the hallmark functions necessary for angiogenesis, tumor formation, and metastasis [2]. Targeting of components of the tumor microenvironment or cancer cells is currently a considerable focus of research interest. In particular, angiogenesis and inflammatory pathways are well-characterized targets for inhibition in hepatocellular carcinoma HCC therapy. For instance, sorafenib, a multiple kinase inhibitor, is one of the most effective suppressors of cell growth and angiogenesis in patients with late-stage HCC.

HCC is among the most common and aggressive human malignancies worldwide. A number of contributory mechanisms to accelerated tumor formation have been proposed to date, including telomere dysfunction and alterations in the microenvironment that induce cell proliferation [3,4]. Another important factor underlying poor prognosis of HCC is the high rate of tumor metastasis. The aggressive nature of the disease highlights the urgent need to identify patients at risk in advance and develop novel targeted agents for successful intervention [5]. Metastasis is a complex process regulated by multiple intrinsic and extrinsic cellular factors. Improved understanding of the associated molecular mechanisms should aid in the development of effective metastasis-targeted therapies and improvement of overall prognosis of patients with HCC [6].

The traditional concept of gene function in molecular biology is the central dogma explaining protein-coding genes (DNA→mRNA→protein). Notably, however, less than 2% of the mammalian genome encodes protein with >90% representing noncoding RNA (ncRNA) [7]. Accumulating evidence has demonstrated the significance of ncRNAs in the regulation of multiple major biological functions controlling development, differentiation, metabolism, cell growth and tumor progression [8]. In general, ncRNAs are classified into two groups based on length, designated small ncRNA and long ncRNA (lncRNA). Small ncRNAs include microRNA (miRNA), transfer RNA (tRNA) and some ribosomal RNA transcripts. MiRNAs are small (~22 nt) non-coding transcripts [9,10] that regulate gene expression at the post-transcriptional or translational level and thereby modulate physiological functions, such as cell growth, migration, invasion, sphere formation and metastasis [11]. Moreover, miRNAs have the ability to regulate hundreds of target genes simultaneously and thus control multiple signaling pathways [12]. Several lines of evidence have demonstrated differential expression of miRNAs, such as miR-155 [13], miR-34a [14] and miR-26 [15], in stromal cells of the tumor microenvironment and their contribution to liver cancer formation.

**Figure 1:**
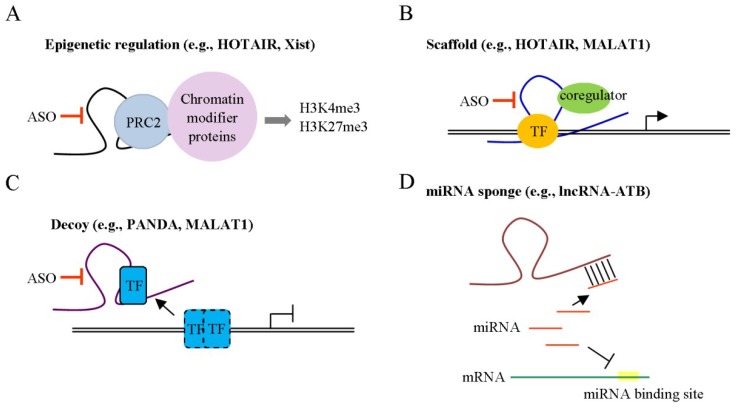
Different mechanisms of action of long non-coding RNAs (lncRNAs). lncRNAs mediate functions by regulating gene expression via diverse molecular mechanisms. (**A**) lncRNAs associate with chromatin-modifying complexes to modulate epigenetic modifications. (**B**) lncRNAs interact with transcriptional factors (TF) or coregulators to regulate gene expression. (**C**) lncRNAs sequester TFs away from chromatin to regulate gene expression. (**D**) lncRNAs serve as a sponge and interact with miRNAs to suppress miRNA–mediated effects. Antisense oligonucleotides (ASO) target lncRNAs, which associate with modulators that translocate to the nucleus, potentially providing a mechanism for targeting these pathways.

lncRNAs are a class of non-protein coding transcripts greater than 200 nucleotides in length [16] frequently dysregulated in various cancers, which also play multiple roles in biological processes, such as proliferation, apoptosis, metastasis and metabolism [8,17]. These transcripts regulate gene expression through effects on the production, splicing, decay or translation of target mRNAs. Interestingly, lncRNAs are transcribed from intergenic regions, antisense strands, introns, gene regulatory regions (promoters and enhancers), untranslated regions (UTR) and telomeres [18,19] and form RNA–RNA, RNA–DNA or RNA–protein interactions to perform their specific activities. lncRNAs are reported to function as guide, scaffold, signaling and decoy RNAs [20] (Figure 1). Guide lncRNAs, such as X inactive-specific transcript (Xist) and Hox transcript antisense RNA (HOTAIR), regulate gene expression *in cis* or *in trans* through recruiting chromatin-modifying enzymes to specific genomic regions [21,22]. As scaffold lncRNAs, HOTAIR or metastasis-associated lung adenocarcinoma transcript 1 (MALAT1) recruit multiple proteins to form ribonucleoprotein complexes and modulate gene expression [23]. Several signaling lncRNAs, including HOTAIR and regulator of reprogramming lincRNA (linc-ROR), act as molecular signals and integrate with specific signaling pathways [24] while the decoy lncRNAs, for instance, P21-associated ncRNA DNA damage activated (PANDA) and MALAT1, sequester transcription factors away from chromatin and regulate gene expression. Functional small peptides encoded by lncRNAs have been identified that are involved in cellular functions [25]. Increasing evidence suggests that the stability of lncRNAs is regulated by miRNAs. On the other hand, lncRNAs can act as competing endogenous (ce) RNAs and sequester specific miRNAs away from their target genes, consequently inhibiting miRNA-mediated functions [26]. Interplay patterns between lncRNAs and miRNAs appear to be crucial events in cancer progression. Emerging data support the involvement of lncRNAs in tumor-stroma communication, a potentially important event in cancer progression. Recently, Sang et al. [27] demonstrated that lncRNA for calcium-dependent kinase activation (CamK-A) is upregulated in several cancers and involved in regulation of the tumor microenvironment through activation of calcium (Ca^2+^)-mediated effects, consequently promoting macrophage recruitment, angiogenesis and cancer progression.

The main objective of this review is to summarize the basic properties and functional roles of the lncRNA-associated tumor microenvironment in HCC. In particular, we have encapsulated current knowledge on the contribution of hypoxia, cytokine- and exosome-modulated lncRNAs to tumor microenvironments that promote angiogenesis, metastasis and drug resistance, with the aim of providing indicators that may serve as future therapeutic markers for various areas of the tumor microenvironment/lncRNAs.

## 2. Cellular Components of the Tumor Microenvironment

**Figure 2:**
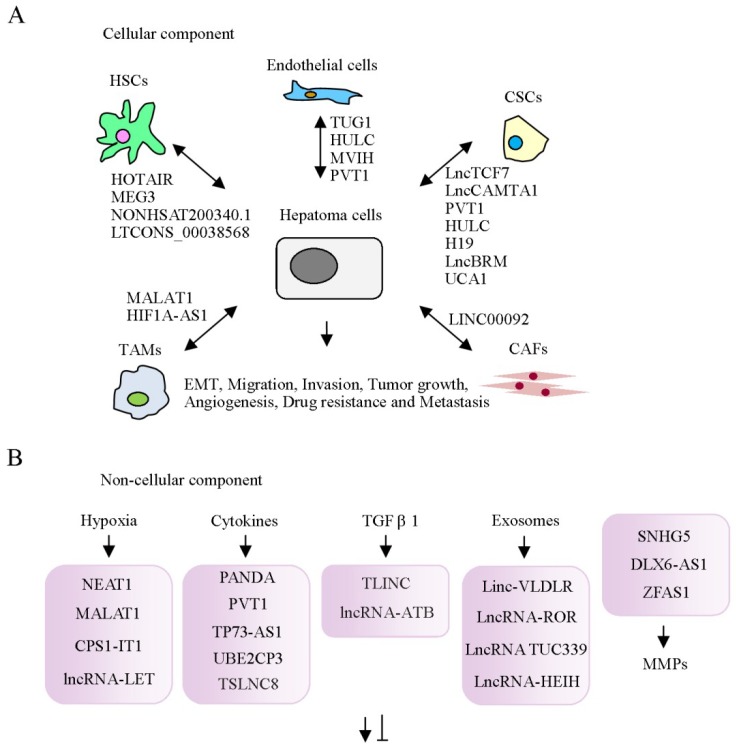
Schematic depiction of significant lncRNAs involved in interactions of hepatoma cells with tumor microenvironment components. (**A**) Cellular components: cancer-associated fibroblasts (CAF), hepatic stellate cells (HSC), tumor-associated macrophages (TAM), endothelial cells and cancer stem cells (CSC) cross-talk with hepatoma cells via multiple lncRNAs, as indicated. (**B**) Non-cellular components: reciprocal regulation of hypoxia, cytokines, TGF-β1, exosomes, matrix metalloproteinases (MMPs), and lncRNAs.

Tumor progression is significantly attributable to surrounding non-tumor cells and non-cellular components secreted from the microenvironment. lncRNA-associated cellular and non-cellular components of the tumor microenvironment in HCC are summarized in Table 1. Cellular components of the tumor microenvironment include cancer-associated fibroblasts (CAF), hepatic stellate cells, tumor-associated macrophages (TAM), endothelial cells, cancer stem cells (CSC), and other immune factors that play crucial roles in inflammation and immunosuppression (Figure 2A) [28,29]. Secreted non-cellular components, including growth factors, cytokines, extracellular matrix proteins and metabolites [30,31], are also crucial in shaping tumor phenotypes and drug responses (Figure 2B). The cellular components are described below.

### 2.1. Cancer-Associated Fibroblasts

Several studies have highlighted the importance of cross-talk between cancer cells and CAFs. These molecules induce oncogenic phenotypes through production of various extracellular matrix proteins, growth factors and cytokines [32,33], such as hepatocyte growth factor (HGF), fibroblast growth factor (FGF), epidermal growth factor (EGF) and transforming growth factor β (TGF-β) [34,35,36]. In addition, HCC cells can be co-cultured with CAFs in vitro. CAFs induced by tissue inhibitor of metalloproteinase 1 (TIMP-1) suppress HCC apoptosis through increasing the Bcl-2/BAX ratio in association with SDF-1/CXCR4/PI3K/AKT signaling [37]. Moreover, CAFs recruit regulatory dendritic cells and facilitate their acquisition of a tolerogenic phenotype through interleukin (IL)-6-mediated signal transducer and activator of transcription 3 (STAT3) activation along with upregulation of Treg via secretion of TGF-β in tumor microenvironments [38]. Signals triggered from cancer cells to CAFs promote tumor survival via these immunosuppressive phenotypes. The lncRNA, LINC00092, is upregulated in ovarian cancer and correlated with poor prognosis. LINC00092 is induced by CAF-secreted CXCL14 and enhances cell metastasis through modulation of phosphofructo-2-kinase/fructose-2 and 6-biphosphatase 2 (PFKFB2) expression [39]. These findings suggest that ovarian cancer cells and CAFs form a positive feedback loop driving glycolysis and tumor progression. The traditional anti-tumor approach involves targeting of epithelial cancer cells. An alternative effective strategy to inhibit tumor formation would be to target CAFs and their communication networks, such as lncRNAs.

### 2.2. Hepatic Stellate Cells

During liver injury, HSCs undergo an important phenotypic change to become myofibroblasts that promote cell growth ability and induce alpha smooth muscle actin (α-SMA) and α-1 collagen expression [40]. Activated HSCs are responsible for production of cytokines, chemokines, growth factors and the extracellular matrix (ECM) [41]. Additionally, these cells penetrate the stromal environment of tumors and coexist with tumor sinusoids, fibrous septa and capsules. Cells treated with conditioned medium from HSC show enhanced growth and migration though modulation of NF-κB and extracellular-regulated kinase (ERK) pathways in vitro [42]. Bian et al. [43] reported a novel mechanism for epigenetic regulation in liver fibrogenesis involving lncRNA-lncRNA interactions. HOTAIR expression was shown to be significantly upregulated in CCl_4_-treated mouse models, human fibrotic liver and activated HSCs. HOTAIR, a component of the polycomb repressive complex 2 (PRC2) complex, controls H3K27me3 modification of chromatin at the promoter region of maternally expressed gene 3 (MEG3) and functions as a competing endogenous RNA (ceRNA) mediating repression of MEG3 via different pathways potentially attributable to localization in HSCs. This is an interesting finding, as it was thought up to now that this lncRNA is switched “on” or “off” in a manner dependent on another lncRNA. Mediation of this control through lncRNAs associated with epigenetic regulators provides an additional level of HSC activation and liver fibrogenesis. Li and co-workers analyzed the expression profiles of lncRNAs in HSC myofibroblasts to ascertain their potential regulatory roles in HSC activation and quiescence and hepatic fibrosis development. The key lncRNAs that could serve as therapeutic targets for suppression of liver fibrosis progression and their regulatory mechanisms were consequently determined. For example, the group reported that NONHSAT200340.1 targets FGF2 to regulate activation of hHSCs via c-Jun N-terminal kinases (JNK) signaling. Another lncRNA, LTCONS_00038568, was shown to target netrin-4 (NTN4) and modulate liver fibrosis through inhibition of epithelial-mesenchymal transition (EMT) [44].

### 2.3. Tumor-Associated Macrophages

The anti-tumor response within the HCC microenvironment is impaired due to immune suppression through the activities of tumor-associated macrophages (TAM) [45]. Intercellular communications between tumor and stromal cells via TAMs play a crucial role in hepatoma [46]. TAMs, mainly comprising the infiltrating leukocyte population, are important for tumor progression. These cells are localized in the stromal component of the tumor mass and polarized to active status [46,47]. Specifically, M2-like TAMs act through the STAT3 signaling pathway and are involved in regulating angiogenesis and metastasis during HCC progression [48]. A number of cytokines, such as IL-4 and IL-10, expressed in the tumor microenvironment trigger TAM polarization to M2-type cells. M2-type TAM expresses a distinctive set of cytokines, including IL-10, and the chemokines CCL17, CCL22 and CCL24, inducing Treg association and inactivation of the Th2 polarized immune response. On the other hand, M2 macrophages are reported to induce vascular endothelial growth factor (VEGF) expression and promote tissue repair and angiogenesis. Kupffer cells are liver-specific TAMs capable of impairing the immune response mediated by T-cell CD8^+^ through association with programmed death 1 (PD1) and programmed death ligand-1 (PD-L1) [49,50]. Huang et al. [51] demonstrated that knockdown of MALAT1 in TAM represses cell growth, migration and invasion of thyroid cancer cell line and reduces angiogenesis. Moreover, these effects were attenuated by overexpression of FGF2. In addition, HIF1A-AS1 was shown to be upregulated by TNF-α via promoting caspase 3 expression in Kupffer cells. Cell apoptosis was enhanced by TNF-α but suppressed upon knockdown of HIF1A-AS1 [52]. These findings support the utility of strategies aimed at modulating the expression of dysregulated lncRNAs in TAMs to facilitate repression of pro-tumorigenic properties.

### 2.4. Endothelial Cells

Endothelial cells are responsible for supporting blood vessel formation and tumor neovasculature. These cells have multiple functions and participate in various molecular signaling pathways in HCC and normal tissues. Several angiogenic receptors, such as C-X-C chemokine receptors (CXCR), epidermal growth factor receptor (EGFR), vascular endothelial growth factor receptor (VEGFR) and platelet-derived growth factor receptor (PDGFR), have been expressed in vitro. Interactions of ligands with these receptors activate signal transduction pathways that trigger survival, proliferation and invasion of endothelial cells [53]. Moreover, tumor-associated endothelial cells display high TGF-β1 and CD105 expression. TGF-β1 acts as a chemoattractant for CD105-expressing endothelial cells that promote angiogenesis [54]. Notably, CD105^+^ endothelial cells from HCC display features of increased angiogenesis activity with higher resistance to chemotherapeutic agents and angiogenic inhibitors [55]. Previously, knockdown of taurine up-regulated gene 1 (TUG1) led to remarkable suppression of tumor-induced endothelial cell proliferation, migration and angiogenesis in vitro [56]. Similar results were obtained in xenograft mouse models. Highly upregulated in liver cancer (HULC), an oncogenic lncRNA, is significantly expressed in HCC [57]. Expression levels of these molecules are correlated with those of sphingosine kinase 1 (SPK1), VEGF and endothelial cell-specific molecule 1 (ESM1) in tumor tissues. Overexpression of HULC promotes tumor angiogenesis, which is blocked in SPK1-depleted cells. Conversely, its knockdown suppresses angiogenesis, tumor cell proliferation and invasion. Furthermore, HULC acts as a ceRNA to inhibit miR-107-mediated suppression of E2F1 and induces angiogenesis, both in vitro and in vivo. Inhibition of E2F1 promotes SPK1 transcription. lncRNA associated with microvascular invasion in hepatocellular carcinoma (MVIH) is encoded in the intron of ribosomal protein S24 (RPS24) gene. MVIH is highly expressed in HCC and positively correlated with tumor growth and intrahepatic metastasis [58] via activation of angiogenesis in mouse models. Phosphoglycerate kinase 1 (PGK1) has been shown to interact with MVIH using the RNA pulldown assay. Moreover, MVIH overexpression is associated with inhibition of PGK1 secretion. PGK1 secreted by tumor cells inhibits angiogenesis and exerts a negative impact on tumor growth and metastasis. A study by Zheng et al. [59] demonstrated that lncRNA-plasmacytoma variant translocation 1 (PVT1) promotes growth, migration and tube formation of endothelial cells. PVT1 inhibits miR-26b activity and induces connective tissue growth factor and angiopoietin 2 expression. Accordingly, the term “Angio-lncRs”, signifying regulation or association with angiogenesis, has been coined [60]. The finding that angiogenesis can be directly or indirectly regulated by lncRNAs further supports the targeting of these molecules to improve angiogenesis-mediated outcomes.

### 2.5. Association between lncRNAs and Cancer Stem Cells

Cancer exists as a heterogeneous population of cells. The different cell types within the population have distinct phenotypic and functional properties, thus limiting therapeutic efficacy. CSC or Tumor-Initiating Cell (TIC) concepts provide an alternative explanation for the failure of existing therapies. Accumulating evidence suggests that CSCs are the root of cancers and responsible for metastasis and resistance to traditional therapies [61,62]. CSCs can self-renew and have pluripotent capacity [63]. CSCs or TICs have been identified in multiple cancer types, including liver cancer. Based on their unique characteristics, CSCs are proposed as critical promotors of tumor initiation, development, metastasis and recurrence. CSC proliferation is regulated by various extrinsic factors derived from the cell microenvironment. For instance, HOTAIR is reported to enhance human liver CSC growth through inhibiting associations of P300, CREB and RNA pol II with the SETD2 promoter region, leading to suppression of SETD2 phosphorylation and expression [64]. lncTCF7 is another critical participant in the regulation of CSC maintenance and renewal in HCC [65] that contributes to cancer progression and development. This lncRNA promotes expression of its target gene, TCF7, through enhancing interactions between the SWI/SNF complex and the TCF7 promoter for transcription. Both lncTCF7 and TCF7 are involved in mediating sphere formation of liver cancer cells, highlighting their importance in CSCs. lncRNA-calmodulin binding transcription activator 1 (CAMTA1) is reported to be upregulated in liver CSCs (CD13^+^/CD133^+^ cells), compared with non-CSCs derived from parental Huh7 and HepG2 cells. Additionally, lncCAMTA1 is highly expressed in HCC tissues. Another study showed that functionally, lncCAMTA1 suppresses the promoter activity of CAMTA1 and induces a repressive chromatin structure, HCC cell proliferation and CSC-like properties [66]. Wang et al. [67] demonstrated that overexpression of PVT1 promotes cell proliferation through regulation of cell cycle-related genes and induces a stem cell-like phenotype of SMMC-7721 cells by stabilizing nucleolar protein 2 (NOP2) nucleolar protein. lncRNA-H19 has been identified in exosomes released by CSC-like CD90^+^ cells. Interestingly, higher expression of lncRNA H19 was detected in exosomes derived from CD90^+^ Huh7 relative to parental Huh7 cells. Moreover, lncRNA H19 was shown to induce pro-angiogenic factors, such as VEGF, in human umbilical vein endothelial cells (HUVECs) and promote adhesion of CD90^+^ Huh7 cells to endothelial cells [68]. The lncRNA associating with Brahma (lncBRM) is additionally highly expressed in HCC tumors and CSC-like CD13^+^/CD133^+^ cells. Mechanistically, lncBRM interacts with Brahma (BRM) to regulate the BRG1/BRM switch in the BRG1-associated factor (BAF) complex and induces YAP1 signaling, subsequently promoting sphere formation and self-renewal [69]. Yet another lncRNA, lncSox4, highly expressed in HCC tumors and CD133^+^ TICs, has been shown to promote self-renewal and tumor-initiating ability through association with STAT3 and upregulation of Sox4 [70]. The lncRNA urothelial cancer associated 1 (UCA1) upregulated in HCC enhances proliferation and tumorigenesis of carcinoma cells [71]. Notably, UCA1 is also upregulated in liver CSCs and plays a critical role in governing their growth and differentiation through regulation of multiple pathways. For example, UCA1 facilitates the differentiation of human embryonic stem cells (ESC) into hepatocyte-like cells through modulation of histone modification. Moreover, UCA1 is reported to trigger hepatocyte-like cell transformation through inducing promoter methylation of HULC and chromatin loop formation of the β-catenin promoter-enhancer [72]. Pu et al. [73] further demonstrated that UCA1 enhances c-Myc expression, RB1 phosphorylation and activity of the retinoblastoma protein Su(var)3-9, Enhancer-of-zeste and Trithorax (SET) domain-containing 1A (pRB1-SET1A) complex, in turn, inducing tri-methylation of histone H3 (H3K4me3) involved in prolongation of telomere length. These findings highlight the critical roles of multiple lncRNAs in modulating CSC maintenance and self-renewal.

## 3. Networks of lncRNAs and Non-Cellular Components of the Tumor Microenvironment in HCC

### 3.1. Association between lncRNAs and Hypoxia

Hypoxic conditions and high expression of the key regulator, hypoxia-inducible factor-1 (HIF-1), are common features in advanced cancers [74,75]. Hypoxic conditions in surrounding cells represent a critical step in the tumorigenic process. Indeed, hypoxia facilitates a number of events in the tumor microenvironment that promote metastasis of heterogeneous tumor cells and is significantly positively correlated with aggressive malignant phenotypes. HIF-1 is a heterodimeric complex composed of two transcription factors, HIF-1α and HIF-2α [76], which regulate genes with significant roles in oncogenic pathways, including apoptosis, proliferation, angiogenesis, tumor metabolism and metastasis. A previous study revealed that expression of the lncRNA TUG1 is enhanced under hypoxia and in human hepatoblastoma [56]. Zheng et al. [77] demonstrated high expression of nuclear paraspeckle assembly transcript 1 (NEAT1) in HCC specimens, which promotes epithelial-mesenchymal transition (EMT), migration and invasion capacities of tumor cells by stimulating HIF-2α activity. Luo and co-workers showed a positive correlation between expression of MALAT1 expression and HIF-2α in HCC tissues [78]. Moreover, arsenite promotes MALAT1 and HIF-2α expression in hepatoma cells. MALAT1 is reported to enhance HIF-2α activity through inhibition of von Hippel-Lindau (VHL) protein-mediated HIF-2α ubiquitination and degradation. Conversely, MALAT1 is regulated by HIF-2α via a feedback loop, supporting the co-involvement of MALAT1 and HIF-2α in HCC. Wang and colleagues identified a novel tumor suppressor lncRNA, CPS1 intronic transcript 1 (CPS1-IT1), with low expression in HCC [79,80]. Overexpression of CPS1-IT1 reduced HIF-1α activity and consequently suppressed EMT progression and HCC metastasis, both in vitro and in vivo. Another lncRNA, Low expression in Tumor (termed lncRNA-LET), is additionally downregulated in HCC [81]. lncRNA-LET is suppressed by hypoxia-induced histone deacetylase 3 through reducing histone acetylation-mediated modulation of its promoter region. Knockdown of lncRNA-LET is a key step in stabilization of nuclear factor 90 protein, which leads to hypoxia-induced cancer cell invasion. HIF-1 and its downstream effectors have been identified as potential targets for cancer therapy. However, owing to the complexity of the hypoxia signaling pathway, inhibition of HIF-1α activity presents a considerable challenge. Recent establishment of the involvement of lncRNAs in hypoxia response in cancers provides further evidence of their potential utility as therapeutic targets.

### 3.2. Association between lncRNAs and Cytokines

Cytokines are major target molecules in a number of inflammatory conditions, with targeted therapies for TNF-α, interferon (IFN), and IL-17 already in clinical use [82,83,84]. Accumulating studies support the involvement of cytokines in hepatocarcinogenesis. A number of investigations have focused on determining whether cytokine expression is correlated with disease progression in tumor-adjacent normal tissues and HCC. Cytokines secreted by tumors or stromal cells in the serum and plasma have been assessed for their predictive capacity in HCC [85,86]. The lncRNA, PANDA, is reported to be downregulated in HCC specimens. Unexpectedly, however, overexpression of PANDA appears to enhance HCC proliferation and tumor growth, both in vitro and in vivo. Mechanistically, PANDA suppresses transcriptional activity of the senescence-associated inflammatory factor, IL8, thereby inhibiting cellular senescence [87]. The lncRNA, PVT1, is induced by IFN-α in HCC cells [88]. Depletion of PVT1 leads to enhanced apoptosis and suppression of growth in IFN-α treated cells. Furthermore, PVT1 represses IFN-α induced phosphorylated signal transducer and activator of transcription 1 (STAT1) and interferon-stimulated gene (ISG) transcription through interactions with STAT1. Upregulation of another lncRNA, TP73-AS1, has been documented in HCC tissues and cell lines [89] in association with poorer prognosis and survival. Knockdown of TP73-AS1 leads to suppression of HMGB1, receptor for advanced glycation end products (RAGE) and NF-κB expression and consequent reduction of cell proliferation. miR-200a has been shown to directly bind TP73-AS1 and the 3’UTR of HMGB1 in the 3’UTR luciferase reporter assay. Moreover, miR-200a knockdown promotes HMGB1, RAGE, NF-κB as well as NF-κB-regulated cytokine (TNFα, IL6 and IL-1β) levels. Expression of ubiquitin-conjugating enzyme E2C pseudogene 3 (UBE2CP3) is higher in HCC than adjacent non-tumor tissues and in tissues with high endothelial vessel density [90]. In studies using a co-culture system, UBE2CP3 promoted HUVEC tube formation, proliferation and migration through the ERK/HIF-1α/p70S6K/VEGFA cascade and enhanced VEGFA expression in HCC cell supernatant fractions. Another novel lncRNA, tumor suppressor long noncoding RNA on chromosome 8p12 (termed TSLNC8), is frequently deleted or downregulated in HCC tissues [91]. Overexpression of TSLNC8 is associated with significant suppression of growth and metastasis, both in vitro and in vivo. TSLNC8 has been shown to modulate STAT3 phosphorylation levels (Tyr705 and Ser727) and transcriptional activity through competitive interactions with transketolase and STAT3, resulting in inactivation of the IL-6/STAT3 signaling pathway in HCC cells. Modulatory roles of lncRNAs in cytokine gene expression are well documented, generating significant research interest in the utility of lncRNAs in therapeutic targeting.

**Figure 3:**
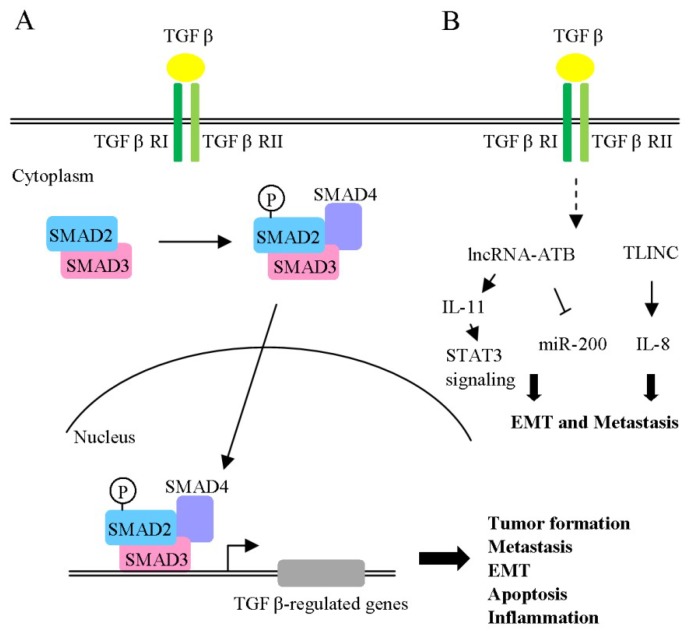
Schematic depiction of the TGF-β signaling pathway. (**A**) TGF-β binds to Type I (TGF-β RI) and Type II receptor (TGF-β RII), whereby TGF-β RII phosphorylates and activates TGF-β RI. Transcriptional factors Smad2 and 3 (Smad2/3) are phosphorylated by TGF-β receptors and associated with Smad4. Activated Smad complexes translocate into the nucleus and regulate target gene transcription. (**B**) lncRNA-ATB and TINC are induced by TGF-β. The downstream molecules regulated by these lncRNAs are depicted. P: phosphorylation.

TGF-β binds to type I and type II receptors (TGF-β RI and TGF-β RII) at the cell surface. Activated TGF-β receptors induce phosphorylation of downstream signal transducer R-Smad (receptor-activated Smad: Smad2 and Smad3). Phosphorylated R-Smads, in turn, associate with Smad4 (Co-Smad) to form a trimeric Smad complex, which translocates into the nucleus and regulates target gene expression (Figure 3A). TGF-β plays a complex role in tumor progression, in particular, liver fibrogenesis and hepatocarcinogenesis [92,93], and is upregulated in HCC tissues and peri-neoplastic stroma. Interestingly, TGF-β1 is known to exert dual effects during HCC progression [94]. In the early stages, the cytokine acts as a tumor suppressor with anti-proliferative effects and stimulation of apoptosis signals. Notably, blockage of cell proliferation is mediated by cyclin-dependent kinase inhibitors and suppression of c-Myc-mediated functions. The tumor suppressor activity of TGF-β is also exerted through suppression of tumor stroma mitogens and tumorigenic inflammation. Conversely, TGF-β plays an oncogenic role, promoting tumorigenicity via several mechanisms, including stimulatory effects on cell migration, angiogenesis and metastasis. TGF-β is proposed to contribute to EMT through downregulation of E-cadherin (epithelial marker) and upregulation of Snail (mesenchymal marker). Another previous report showed that TGF-β1 enhances miR-181b expression, promoting growth, survival, migration and invasion of HCC cells [95]. Similarly, miR-23a, miR-24 and miR-27a appear to enhance tumor cell survival in HCC [96]. A novel lncRNA, designated TGF-β-induced long non-coding RNA (TLINC), was further identified as a target induced by TGF-β in both hepatic and non-hepatic cells (Figure 3B) [97]. Interestingly, expression of two TLINC isoforms (long and short) was associated with the epithelial and mesenchymal phenotype, respectively. The long isoform of TLINC was positively correlated with metastatic phenotype and increased levels of proinflammatory cytokines (IL-8). TLINC was additionally detected in both epithelial and stromal cells and identified as a tumor marker. Another lncRNA (lncRNA-ATB) is reported to be induced by TGF-β1 [98]. Clinically, lncRNA-ATB was overexpressed in HCC specimens and enhanced EMT and metastasis through modulation of the ZEB1/ZEB2/miR-200 cascade. In addition, lncRNA-ATB increased colonization of migrating cells by triggering the IL-11/STAT3 signaling pathway. Mechanistically, IL-11 mRNA stability was enhanced via lncRNA-ATB interactions, which, in turn, facilitated IL-11 secretion, suggesting a role in phosphorylation of STAT3 (Figure 3B). This autocrine regulatory mechanism promotes cell survival and colonization to distant organ sites. The collective findings indicate that several lncRNAs are regulated by TGF-β and play important roles in TGF-β-mediated effects on EMT, migration and invasion.

### 3.3. Regulation of the Tumor Microenvironment by lncRNAs in Exosomes

Exosomes have recently been identified as critical mediators of cell-to-cell communication in cancer progression through transfer of RNA and proteins to neighboring or distant cells [99]. The compositions of exosomes differ depending on cell type, physiological and pathological conditions. Previous studies have reported that exosomes are 40–150 nm in diameter and exist in both normal and tumor cells. Exosomes play an important role in crosstalk between tumor and stromal cells and deliver specific molecules to target cells through endocytosis and phagocytosis [100,101]. Moreover, exosomes fuse with membranes of target cells to deliver components into cells. According to their origin in the tumor microenvironment, exosomes can be classified as tumor cell or stromal cell secretions. Signal transduction between these cells in the tumor microenvironment is significantly involved in regulation of cell invasion, metastasis, drug resistance and cancer. The exoRBase database (http://www.exoRBase.org) contains information on circular RNAs (circRNA), lncRNAs and mRNAs derived from RNA-seq data analyses of human blood exosomes [102]. To date, few lncRNAs, such as lincRNA-VLDLR, ROR, and TUC339, have been detected in circulating HCC extracellular vesicles [103,104,105]. Takahashi et al. [88] demonstrated that anticancer drugs induce linc-VLDLR expression in cells as well as extracellular vesicles (EV) released from these cells. Notably, chemotherapy-induced HCC cell death was repressed upon incubation with EV. These effects were reduced upon knockdown of linc-VLDLR cell lines. Another lncRNA involved in HCC resistance against microenvironmental conditions is lncRNA-ROR, which promotes EMT, cancer stem cell maintenance and tumorigenesis. While overexpression of lncRNA-ROR has been established in normal hepatocytes, its selective enrichment within extracellular vesicles is correlated with TGF-β-dependent HCC cell chemoresistance and knockdown shown to increase chemosensitivity. The lncRNA TUC339 is significantly expressed in extracellular vesicles derived from HCC cells and implicated in tumor growth, cell adhesion and cell cycle progression. Recently, Sun and co-workers reported the presence of higher levels of LINC00161 in serum exosome and urine samples from HCC patients, compared to controls [106]. Another group demonstrated increased lncRNA-HEIH expression in serum and exosomes of HCV-related HCC [107]. Clearly, lncRNAs are involved in exosome-mediated functions and may, therefore, serve as potential targets for therapeutic interventions.

### 3.4. Extracellular Matrix (ECM) and Matrix Metalloproteinases within the Microenvironment

Extracellular matrix (ECM) is produced by stromal cells in the microenvironment. The components of ECM, including laminin, collagens, fibronectin and proteoglycans, are associated with altering the phenotype and function of HCC cells. ECM production and reorganization can promote tumor cell proliferation and invasion and alter gene expression in different stromal cell and cancer cell types, leading to tumor progression [108]. Accumulating evidence supports the view that extracellular proteinases, such as matrix metalloproteinases (MMP), mediate many of the changes in the microenvironment during tumor progression. The complexity of the tumor microenvironment triggers regulatory cascades that determine the functions of the diverse MMPs expressed. Proteolytic cleavage of MMPs is regulated at different levels, including gene expression, conversion from the pro- to active form and specific inhibitors. Conversion of the pro-form (pro-MMP) into its active form is regulated by proteinases, such as furin and plasminogen, a critical step for MMP activity [109,110]. MMP activity is additionally modulated by lncRNAs. For example, functional knockdown of small nucleolar RNA host gene 5 (SNHG5) highly expressed in HCC is reported to induce apoptosis and suppress cell cycle progression, growth and metastasis in hepatoma cell lines whereas its overexpression has the opposite effects. Importantly, SNHG5 knockdown led to inhibition of MMP-2 and MMP-9 that are closely related to metastasis [111]. Zhang et al. [96] demonstrated upregulation of the lncRNA DLX6-AS1 in HCC samples, which was correlated with poor prognosis of HCC patients. Knockdown of DLX6-AS1 suppressed cell growth, migration and invasion, both in vitro and in vivo. MiR-203 targeting the 3’UTR region of DLX6-AS1 was negatively correlated with DLX6-AS1 expression. Data from the 3’UTR luciferase reporter assay further revealed that miR-203a targets MMP-2 mRNA. These collective findings support an oncogenic role of DLX6-AS1 in clinical specimens and cellular experiments, indicative of the involvement of a potential DLX6-AS1/miR-203a/MMP-2 pathway in tumorigenesis [112]. lncRNA ZFAS1 gene amplification observed in HCC is positively correlated with hepatic invasion and metastasis through modulation of the miR-150/ZEB1/MMP14/MMP16 cascade [113]. These lines of evidence clearly support regulation of MMPs by lncRNAs. Direct inhibitors of MMP have been developed in previous studies. Another strategy to effectively achieve MMP inhibition is targeting specific lncRNAs to reduce MMP activity.

### 3.5. Metabolites and the Tumor Microenvironment

Interestingly, secreted metabolites function in decisions on the phenotypic diversity of cells in the tumor microenvironment. Accumulating evidence supports the theory that secreted metabolites act as tumor morphogens that shape unique tumor heterogeneity. CAFs residing in the tumor microenvironment promote cancer cell growth by producing metabolites, such as lactate, fatty acid and amino acids [114]. Earlier, liver-specific miR-122 expression was shown to be reduced in HCC. Moreover, overexpression of miR-122 led to inhibition of HCC formation in an animal model. Mechanistically, miR-122 reduces lactate production and promotes oxygen consumption through inhibition of pyruvate kinase M2 (PKM2) expression [115]. Consistent with this finding, another study demonstrated that miR-122 mediates inhibition of PKM2 protein expression by directly targeting its 3’UTR region in HCC [116]. Current knowledge, along with data obtained from high-throughput tools, such as RNA-seq and metabolomic analyses, indicate that interplay between metabolites and ncRNAs plays a crucial regulatory role in cancer progression.

## 4. lncRNAs as Novel Targets for HCC Therapy

Traditional cancer treatment is dependent on average patient response. However, treatments that can be successfully applied for some patients may not be effective for others. An emerging approach for disease treatment and prevention, known as precision medicine, has gained significant attention as a means to improve treatment outcomes. Precision medicine is based on knowledge of individual variabilities in the genes, epigenetic profiles and environments for each patient [117]. This theory assumes that a disease is caused by specific genes or molecular pathways. Individual diseases or cancers are therefore tightly associated with variations in genes and downstream signaling pathways. Identification of effective drug-specific targets for tumor cells that do not adversely affect normal cells is a considerable challenge in therapy. Therefore, effective treatments need to target specific molecules that act as drivers in development of cancer. Previously, miRNAs have been identified in biological fluids of patients, such as urine and blood [118], which act as potential biomarkers in diagnosis and prognosis of cancers. More recently, lncRNAs have been highlighted as potential candidates for biomarkers and precision medicine targets in cancer due to their specific expression patterns in tumor cells [119,120]. In addition, lncRNAs are detectable in biological fluids of patients and may, therefore, be applied as noninvasive markers for clinical analysis.

DNA methyltransferase (DNMT) and histone deacetylase (HDAC) inhibitor drugs [121] are commonly used to treat various cancer types. However, these agents that act via epigenetic regulation are nonspecific and should be delivered via venous injection. Notably, lncRNAs regulate similar genes or functions through epigenetic mechanisms and are considered drug targets with a lower incidence of side-effects and improved specificity. Drug resistance is an important issue that limits effective cancer treatment. lncRNAs involved in drug resistance through modulation of drug transporter expression, oncogenic survival signaling pathways, cell cycle, and apoptosis have been identified. As mentioned above, TGF-β-dependent chemoresistance is regulated by lincRNA-ROR in HCC [105]. Another study by Li and co-workers demonstrated that lncRNA Activated in RCC with Sunitinib Resistance (lncARSR) is involved in doxorubicin resistance through regulation of the PTEN/PI3K/Akt pathway in HCC [122].

lncRNA-specific therapeutic approaches target lncRNA-mediated functions and pathways through gene silencing and structure disruption mechanisms. In fact, a single lncRNA can regulate several protein coding genes and pathways. In such situations, manipulating individual lncRNAs can modulate multiple genes and their functions. The currently available strategies are outlined below. For instance, expression of lncRNA could be suppressed with short interfering RNAs (siRNA), short hairpin RNAs (shRNA), antisense oligonucleotides (ASO), locked nucleic acid (LNA) gapmeRs and clustered regularly interspaced short palindromic repeat-associated nuclease 9 (CRISPR/Cas9) systems. However, the knockdown efficiency of these genes is dependent on their localization. Knockdown of nuclear lncRNAs is successfully achieved through ASOs whereas siRNAs work most effectively in the cytoplasm. Modulation of lncRNA expression with CRISPR interference through guide RNAs (gRNA) reduces the possibility of off-target effects and allows suppression independent of the subcellular location of the target. Although several lines of evidence provide convincing results regarding lncRNA-mediated functions and their utility as therapeutic targets, their use in vivo is extremely challenging owing to poor conservation of lncRNAs across species. The mechanisms of action of lncRNAs, such as recruiting/binding partners, in animal models, differ from in vitro experimental findings. Notably, lncRNAs differ from protein-coding genes in several ways that require consideration in analyzing their effects or therapeutic potential. The complex structures formed by lncRNA-lncRNA, lncRNA-protein and lncRNA-DNA molecules may provide new strategies to disrupt these interactions. Moreover, expression of lncRNAs is tissue- or cell-type specific. Finally, current knowledge of non-coding gene functions highlights the combinatorial nature of their actions, which involve complex interactions incorporating multiple associated effectors. Recently, Amodio et al. [123] demonstrated that inhibition of MALAT1 by LNA gapmeR ASO suppresses multiple myeloma cell growth and induces apoptosis in vitro and in vivo. In addition, a peptide nucleic acid (PNA)-targeting approach for lncRNA was established. This strategy was successful in blocking interactions of HOTAIR with EZH2, leading to suppression of HOTAIR-EZH2 activity and increased chemotherapy sensitivity [124]. Notably, the phenotypes of target lncRNAs could differ depending on the “tissue context”, which should be analyzed to achieve optimal therapeutic responses. 

## 5. Conclusions

In the current article, lncRNAs in the tumor microenvironment involved in the regulation of tumor growth, angiogenesis and metastasis are comprehensively listed in Table 1. While several genetic, epigenetic, transcriptional and translational dysregulation processes collectively contribute to HCC, existing knowledge regarding the signaling pathways that influence HCC is incomplete. Recent extensive characterization of lncRNAs as initiators or diagnostic markers of HCC has highlighted their utility as important regulators in HCC progression. Due to their functional roles as either tumor suppressors or oncogenes involved in diverse cellular networks, lncRNAs may be developed as a molecular tool suitable for application in therapeutic and clinical strategies for HCC. The collective results support multiple interactions between the tumor microenvironment and lncRNA networks that drive cancer cell survival, resistance to therapy and metastasis. Increasing evidence of dysregulated lncRNAs in various malignant tumors that may serve as potential biomarkers has been documented. Liver cancer presents a complex model to investigate the relationship between the microenvironment and tumor development. Improved knowledge of these interactions is therefore essential to identify potential prognostic/predictive biomarkers and successfully develop novel targeted therapies.

## Figures and Tables

**Table 1:** Tumor microenvironment-related lncRNAs and their potential mechanisms in hepatocellular carcinoma (HCC).

Gene Name	Principal Functions	Molecules and Signaling Pathways Involved ^a^	Expression in HCC	Prognostic MARKERS in HCC ^b^	Cellular/Non-Cellular Component ^c^	Regulation Mechanism ^d^	Reference
*TUG1*	Tumorigenesis Angiogenesis	MiR-34a-5p, VEGFA	Up	✓	Both	Hypoxia	[56]
*HULC*	Angiogenesis Cell growth Invasion	MiR-107, E2F1, SPK1, ESM-1 PI3k/Akt/mTOR pathway	Up	✓	Cellular	-	[57]
*MVIH*	Tumor growth Metastasis Angiogenesis	PGK1	Up	-	Cellular	-	[58]
*HOTAIR*	Cell growth	P300, CREB, RNA pol II	Up	✓	Cellular	-	[64]
*lncTCF7*	Tumorigenicity Self-renewal EMT	Wnt signaling, SWI/SNF complex, TCF7	Up	-	Cellular	-	[65]
*lncCAMTA1*	Proliferation CSC-like properties	CAMTA1	Up	✓	Cellular	-	[66]
*H19*	Angiogenesis	Angiogenin, FGF18	Up	✓	Cellular	-	[68]
*lncBRM*	Sphere formation Tumor formation	BRG1/BRM switch, YAP1 signaling	Up	✓	Cellular	-	[69]
*lncSox4*	Self-renewal Tumor-initiating ability	STAT3, Sox4	Up	✓	Cellular	-	[70]
*UCA1*	Proliferation Tumorigenesis	MiR-216b, FGFR1/ERK signaling pathway	Up	-	Cellular	-	[71]
*NEAT1*	EMT Migration Invasion	HIF-2α pathway	Up	✓	Non-cellular	HIF-2α	[77]
*MALAT1*	Transformation	VHL, HIF-2α	Up	✓	Non-cellular	MALAT1/HIF-2α feedback loop	[78]
*CPS1-IT1*	EMT Metastasis	HIF-1α activity	Down	✓	Non-cellular	-	[79,80]
*lncRNA-LET*	Invasion	NF90, HIF-1α, CDC42	Down	-	Non-cellular	HDAC3	[81]
*PANDA*	Proliferation Tumor growth Cellular senescence	IL8	Down	✓	Non-cellular	-	[87]
*PVT1*	Proliferation Apoptosis Cell proliferation Stem cell-like phenotype	STAT1/ISG pathway, NOP2	Up	✓	Both	IFN-α	[67,88]
*TP73-AS1*	Proliferation	HMGB1, RAGE, NF-κB, MiR-200a	Up	-	Non-cellular	-	[89]
*UBE2CP3*	Proliferation Migration Tube formation	ERK/HIF-1α/p70S6K/VEGFA	Up	✓	Non-cellular	-	[90]
*TSLNC8*	Cell growth Metastasis	IL-6/STAT3 signaling pathway, Transketolase	Down	✓	Non-cellular	-	[91]
*TLINC*	EMT	IL8	Up	-	Non-cellular	TGF-β	[97]
*lncRNA-ATB*	Migration Invasion Metastasis	MiR-200, IL-11/STAT3 signaling pathway	Up	✓	Non-cellular	TGF-β1	[98]
*linc-VLDLR*	Chemotherapy	Exosome	Up	-	Non-cellular	-	[104]
*lincRNA-ROR*	EMT CSC maintenance Tumorigenesis	TGF-β-dependent chemoresistance	Up	✓	Non-cellular	-	[105]
*lncRNA-TUC339*	Tumor growth Cell Adhesion Cell cycle	Exosome	Up	✓	Non-cellular	-	[103]
*LINC00161*	Migration Invasion	Serum exosome Urine sample	Up	✓	Non-cellular	-	[106]
*HEIH*	Cell cycle progression	EZH2	Up	✓	Non-cellular	-	[107]
*SNHG5*	Apoptosis Cell cycle Metastasis	MMP2, MMP9, miR-26a-5p, Wnt/β-catenin/GSK3β signal pathway	Up	✓	Non-cellular	-	[111]
*DLX6-AS1*	Cell growth Migration Invasion	MiR-203a/MMP2 pathway	Up	✓	Non-cellular	-	[112]
*ZFAS1*	Metastasis	MiR-150, ZEB1, MMP14, MMP16	Up	✓	Non-cellular		[113]

a: Downstream molecules and signaling pathways involved in lncRNA-mediated functions; b: ✓: lncRNAs acting as prognostic markers in HCC. -: Information is unavailable’ c: lncRNAs related to the cellular or non-cellular component of the tumor microenvironment (Cellular: cellular component, Non-cellular: non-cellular; component, Both: cellular and non-cellular component); d: lncRNAs regulated by upstream transcriptional factor or cytokines, as indicated. -: Information is unavailable.
